# Relationship between obesity and coronary heart disease among urban Bangladeshi men and women

**DOI:** 10.15761/iod.1000112

**Published:** 2015-05-25

**Authors:** Rumana J Khan, Danielle J Harvey, Bruce N Leistikow, KMHS Sirajul Haque, Christine P Stewart

**Affiliations:** 1 Graduate Group in Epidemiology, University of California, Davis, CA, USA; 2 International Centre for Diarrhoeal Disease Research, Dhaka, Bangladesh; 3 Program in International and Community Nutrition, University of California, Davis, CA, USA; 4 Department of Public Health Sciences, University of California, Davis, CA, USA; 5 Department of Cardiology, Bangabandhu Sheikh Mujib Medical University, Dhaka, Bangladesh

**Keywords:** obesity, body mass index, waist circumference, coronary heart disease

## Abstract

The aim of the study was to examine the association of different measures of obesity (body mass index or BMI, waist circumference or WC, waist to hip ratio or WHR and waist height ratio or WHtR) with coronary heart disease (CHD) in a Bangladeshi population. The study included 189 hospitalized CHD cases (133 men and 52 women) and 201 controls (137 men and 68 women). Logistic regression was done to assess the associations between obesity and CHD. The mean age was 53.1 ± 8.3 for men and 51.9 ± 8.4 for women. After adjustment for confounders the odds ratio (OR) of CHD for men was 1.69 (95% CI, 1.24–2.32), 1.94 (95% CI 1.40–2.70), and 1.32 (95% CI, 1.01–2.16) per 1 standard deviation (SD) increase in BMI, WC, and WHtR respectively. The OR for women was 2.64 (CI, 1.61–4.34), 1.82 (95% CI 1.12–2.95), 2.32 (95% CI, 1.36–3.96), and 1.94 (95% CI, 1.23–3.07) per 1 SD increase in BMI, WC, WHtR and WHR respectively. Since both total obesity and abdominal adiposity were associated with development of CHD and since measurement of WC and BMI are inexpensive, both should be included in the clinical setting for CHD risk assessment for this group of population.

## Introduction

Large-scale prospective studies of cardiovascular disease have described a significant, independent relationship between body mass index (BMI) or total obesity and coronary heart disease (CHD) [1–4]. However, it also has been argued that BMI does not adequately reflect body fat distribution, and abdominal obesity, which captures the distribution of fat mass may be an even more important predictor of CHD [5,6]. In epidemiologic settings, as a marker of visceral fat mass or abdominal adiposity, waist circumference (WC; abdominal girth), waist circumference to hip circumference ratio (WHR; waist hip ratio), and ratio of waist circumference to height (WHtR; waist to height ratio) are used to assess CHD risk. It is well established that there are ethnic differences in body fat distribution and in relationships of different obesity measures to CHD or to CHD risk factors [7,8]. Asians and South Asians generally have a higher percentage of body fat than white people of the same age, sex, and BMI. This contributes to the higher prevalence of cardiovascular risk factors at lesser degrees of obesity [9–11]. Because of greater predisposition of abdominal obesity and visceral fat, they also can have increased abdominal obesity with a lower BMI [12,13]. Thus the relationship between these anthropometric measures and CHD in south Asian population can be complex. CHD has become the major killer for adults of South Asian region including Bangladesh and it is projected that over the next 10 years, the rates of CHD will rise substantially [14–16]. The prevalence of obesity had also substantially increased in Bangladesh in last few decades [17–20]. Studies have rarely attempted to document the association between total or abdominal obesity and CHD in Bangladesh and to the best of our knowledge; such data are also limited in the South Asian context. In this study we evaluate the association of different measures of obesity (BMI, WC, WHR and WHtR) with CHD in an urban Bangladeshi population.

## Materials and methods

### Study participants

This hospital-based, prospective case-control study was conducted at Bangabandhu Sheikh Mujib Medical University (BSMMU) hospital in Dhaka, the capital city of Bangladesh. 30 to 70 year old CHD patients, hospitalized with their first diagnosed incident of non-fatal myocardial infarction (either first incident of acute myocardial infarction or a first incident of angina pectoris) were included as cases within 7 days of their admission. Acute myocardial infarction was confirmed by clinical examination, plus either electrocardiogram changes (new pathologic Q waves or 1-mm ST elevation in any 2 or more contiguous limb leads or a new left bundle branch block or new persistent ST-T wave changes diagnostic of a non–Q-wave myocardial infarction) or elevated cardiac enzyme measurement (creatine phosphokinase-MB enzyme or Troponin I) [21,22]. Angina pectoris was confirmed by clinical examination and: 1) coronary angiogram (≥ 50% occlusion in ≥1 of 3 main coronary arteries), or 2) positive exercise stress test (if no angiographic data were available); or 3) electrocardiographic changes at rest (if no angiographic, or exercise stress data were available) [21,22]. Cases were excluded if the diagnosis was made more than two weeks prior to hospitalization, or if they had pre-existing CHD or stroke. Patients with history of any kind of severe chest pain, pregnant patients and patients with any kind of gastrointestinal disorders (e.g. peptic ulcer disease, carcinoma of esophagus, stomach, small and large intestine) were also excluded. The controls were individuals who came to the same hospital to treat ailments that were not related to obesity. Controls were obtained from noncardiac (ophthalmology; ear, nose, and throat; dermatology; orthopedics; general surgery; gynecology) outpatient clinics or inpatient wards. The same exclusion criteria used for cases were applied for control selection. To increase the efficiency of the study, controls were frequency matched by age (within 10 year) and sex. Controls were recruited after collecting data from all the cases. In total, there were 189 cases and 201 controls for the current analysis. The study protocol was approved by the institutional review board of University of California Davis and the ethical review committee of BSMMU.

### Data collection

A structured questionnaire was administered in-person to both cases and controls in the same manner by trained research assistants. The questionnaire included participant demographics (age, sex, marital status and residence type), socioeconomic status (education of the participant and monthly household income), and lifestyle (tobacco use, physical activity level and personal history of CHD risk factors). Physical measurements including weight, standing height, and circumferences of waist and hip, was carried out in the same manner in cases and in the controls according to standard protocol by trained assistants. During the measurements, participants were asked to be relaxed with arms held loosely at sides. Standing height was measured using a stadiometer (Seca 217, Hamburg, Germany) to the nearest 0.1 cm with the participant in bare feet, back against the wall with eyes looking straight ahead. Weight was measured in light clothing using a digital scale, to the nearest 0.1 kg (Adam Equipment CPWplus, Milton Keynes, MK1 1SW, United Kingdom). WC was measured with a non-stretchable standard tape over the unclothed abdomen at the smallest diameter between the costal margin and the iliac crest. Measurement of HC was taken over light clothing at the level of the greater trochanters (usually the widest diameter around the buttock) by a non-stretchable standard tape.

### Analysis

Continuous variables, including age, household income, weight, height, WC, and HC were checked for normality, outliers and missing values. BMI was calculated as weight in kilograms over height in meters squared. WHR and WHtR were calculated as the ratios of WC and HC, and ratios of WC and height respectively. Descriptive statistics were generated separately for male and female participants. Socio-demographic characteristics, anthropometric indices and cardiovascular risk factors for the cases and controls were compared using the chi-square test for categorical variables or independent t test for continuous variables for both males and females. The primary outcome measure for this analysis was CHD (no/yes) evaluated as a dichotomous variable. Logistic regression was used, keeping each of the measurement indices (WC, WHR, WHtR and BMI) as independent variables to examine for their individual associations with CHD. Gender-specific odds ratios (OR) and 95% confidence interval (CI) were calculated for per 1 standard deviation (SD) change in the obesity measurement indices. For relative comparison between total and abdominal obesity, BMI and WC were categorized into quartiles and ORs were estimated for each quartile of BMI and WC keeping first quartile as reference.

The first group of multivariate models to determine the association between the exposures and the outcome were adjusted for participant’s age, education (college level, secondary school, primary school and no schooling), smoking status (never smoker, former smoker, current smoker), physical activity level during leisure time (sedentary to mild versus moderate to strenuous) physical activity level during work (sedentary to mild versus moderate to strenuous), family history of CHD and residence type (urban versus rural). The second multivariable model additionally adjusted for covariates likely to be in the biologic pathway relating obesity to CHD such as self-reported hypertension and diabetes to assess further whether independent effects of different types of obesity measures were mediated by these pathologic mechanisms. We report the results of multiple logistic regressions separately for males and females. The assumptions of logistic regression models were tested by Hosmer and Lemeshow goodness-of-fit test. The index plots for the residuals were also checked for possible deviations.

## Results

Table 1 shows the socio demographic characteristics of the participants. The overall mean age of cases with first CHD was almost the same for men (53.0 ± 8.3) and women (52.6 ± 8.4). The highest proportion of both male and female cases belonged to age group 50–59 years. A higher percentage of male and female CHD cases were from rural areas compared to controls. Male CHD cases were less educated than male controls. About 56.4% (21.1% no education and 35.3% primary education) of the male cases had no or low education compared to only 19.9% (8.1% no education and 11.8% primary education) in the control group (p<0.001). In women, about 92.3% (61.5% no education and 30.8% primary education) of the cases had no or low education, compared to 82.1% (32.8% no education and 49.3% primary education) in the control group (p=0.07).

The distribution of various risk factors between cases and controls is reported in Table 2. Male cases smoked more than the male controls. About 42.1% of the cases were current smokers, while 19.0 % of the controls smoked currently. 28.6% of male cases and 36.5% of female cases had diabetes mellitus compared to 11.8% in male controls and 26.9% in female controls. The difference of this prevalence between cases and controls was significant for males (P <0.001), but not for females. The prevalence of hypertension was higher than diabetes mellitus among the study participants. About 66.9% of male cases and 73.1% of female cases had hypertension compared to 37.5 % in male controls and 25.4% in female controls. The difference between cases and controls was significant for both males and females (p value <0.001). About 26 (19.5%) male cases and 13 (25.0%) of female cases had both hypertension and diabetes mellitus. On the other hand, for controls, 8 (5.9%) males and 11 (16.4%) females had them both. The mean values of different measures of obesities (BMI, WC, WHR and WHtR) were significantly higher in cases than in controls for both sexes (p<0.05), except for WHR and WHtR in males.

Table 3 provides the overall odds ratios for different measures of obesity. After adjustment for age, smoking, leisure time physical activity, work time physical activity, education status, family history of CHD and residence type, the OR of CHD for males was 1.69 (95% CI, 1.24–2.32) per 1 SD increase in BMI, 1.94 (95% CI 1.40–2.70) per 1 SD increase in WC, and 1.32 (95% CI, 1.01–2.16) per 1 SD increase in WHtR. The risk estimates for WHR in men were not statistically significant. On the other hand, after adjusting for the same factors, all of the obesity indicators remained statistically significant for female participants. The risk estimates however, were mostly larger in magnitude and had wider CIs. The OR for CHD was 2.64 (CI, 1.61–4.34) per 1 SD increase in BMI, 1.82 (95% CI 1.12–2.95) per 1 SD increase in WC, 2.32 (95% CI, 1.36–3.96) per 1 SD increase in WHtR and 1.94 (95% CI, 1.23–3.07) per 1 SD increase in WHR. Further adjustment for covariates in the biologic pathway relating obesity to risk of CHD (history of diabetes and hypertension) attenuated the odds ratios for all the measures except for WHR in female where the odds ratio increased slightly (Table 3).

Table 4 and 5 show the unadjusted and adjusted ORs of CHD by quartile of BMI and WC among men and women respectively, with the first quartile in each case serving as the reference category. For men, after adjusting for confounders variables the odds of having CHD was significantly increased by 3.39-fold (95% CI,1.44–8.00) in the second quartile (BMI range 23.16–24.60) and by 4.78 fold (95% CI, 2.0–11.44) in the fourth quartile (BMI range 26.23–31.37) relative to the first quartile. For WC the odds increased significantly by 3.23 fold (95% CI, 1.41–7.37) in the fourth quartile (WC range 92.4 cm–120.00 cm) relative to the first quartile. The second and third quartiles of WC were not significantly different than the lowest quartile. The trend was similar when the intermediary variables were included in the model. With women, CHD risk was significantly increased by more than 9-fold (OR, 9.45; 95% CI, 3.09–33.48) in the fourth quartile of BMI (BMI range 26.33–30.90) relative to women in the lowest quartile. With increasing WC values, the risk of CHD also increased. Women in the third quartile (WC range 84.80 cm–89.00 cm) had a 3.78 fold (95% CI, 1.19–12.09) increased risk of CHD and women in the fourth quartile (WC 89.50 cm–106.00) had a 3.51 (1.03–12.00) increased risk of CHD compared with those with a WC in the lowest quartile. However, the associations with WC diminished and were no longer statistically significant when the intermediary variables were included in the model.

## Discussion

Our results indicated that both general or total obesity (measured in BMI) and abdominal adiposity (measured in WC, WHR or WHtR) were associated with development of CHD. For men, BMI, WC, and WHtR were independently associated with CHD, but WHR was not. On the other hand, for women all four measures were associated after controlling for confounders. When the quartiles were examined across the whole distribution, the association with BMI remained significant even after adjusting for intermediary variables. In contrast, the association became relatively weaker for WC. These observations implied that in our cohort, BMI appeared to predict CHD risk slightly better than central or abdominal obesity.

Several authors have reported the CHD risk associated with BMI, WC, WHR or WHtR and have systematically compared some or all of these indicators to predict CHD [23–28]. The studies from developed countries are mostly prospective in nature. These studies confirm that obesity measured by any index almost always is associated with increased risk of CHD. However, the findings of comparing different measurements of total obesity (BMI) and abdominal obesity (WC, WHR, WHtR) in predicting CHD events have not been consistent. Some have suggested that total obesity rather than abdominal obesity better predicts CHD [27,28], while some investigators have found the reverse to be true [23,26], and still others didn’t find any significant difference [24,25]. Unlike developed countries, in Asian countries, especially in South Asia most of the research done on this subject has used either cross sectional or case control designs [29,30]. The findings also have been based on associations of obesity indices with other CHD risk factors (hypertension, diabetes, dyslipidemia or metabolic syndrome) rather than with an endpoint of CHD itself [31–34]. Studies from India and China suggested that for both sexes obesity measured by high BMI, WC, WHR or WHtR were identical in predicting CHD risk factors [31–34]. In contrast, a global case-control study that had data from South Asia suggested that elevated WHR and high WC were significant predictors for CHD, but BMI was not [29]. However, a prospective Chinese study with CHD as endpoint reported that both WHR and BMI were equally important in predicting CHD risk [35]. This inconsistency in findings could be due to a number of reasons. There can be errors in self-reported measurements in some studies and that can either cause spurious associations or can bias results towards the null. Inadequate or over adjustment of confounders and other cardiovascular risk factors also play a role in determining the nature of this association. Fat distribution and susceptibility to CHD vary by age, sex and ethnicity and can cause these differences in results as well.

Our study showed that obesity measured either as BMI, WC, or WHtR was associated with development of CHD for both men and women independent of other cardiovascular risk factors. The association with BMI appeared to be slightly stronger for both sexes as the association was more consistent across the quartiles than other measures. However, in recent years, BMI has been criticized as a measure of risk because it reflects the total obesity and does not identify fat distribution [36]. Few studies have highlighted that abdominal adiposity is a more important risk factor for cardiovascular disease than is total obesity because intra-abdominal or visceral fat is more metabolically active than subcutaneous fat and accumulation of intra-abdominal or visceral adipose tissue promotes insulin resistance, dyslipidemia and hypertension and thus increases the risk of CHD [37,38]. Some studies have suggested redefining obesity based on WHR or WC instead of BMI [29,39]. But the importance of BMI should not be ignored or be underestimated especially in the South Asian region as people get CHD at a relatively early age than the Western countries and there are reasonable evidence to believe that BMI predicts CHD better than WC or WHR at younger ages [24,35,40]. In the Health Professionals Follow-up study, BMI predicted the risk of CHD better than WHR among young subjects, however, for the elderly, WHR was the better predictor [40]. A recent study with longer follow up of the Health Professionals study and The Nurses’ Health Study also showed that WC predicted CHD risk better than BMI among men and women only above age 60 and BMI was more strongly associated with risk of CHD in the younger than in older participants [24]. This age variation was also reported in a Chinese study where only BMI was associated with CHD risk among women below 55 year of age. However, among older women, WHR was the only independent anthropometric predictor [35]. The precise reason behind this age variation is not fully known. Partially it can be explained by the fact that lean body mass of the body doesn’t vary much in younger adults; and therefore, for them, differences in BMI are likely to reflect differences in fat mass as BMI is a combined measure of both lean body mass and fat mass, adjusted for height. On the other hand, in older people, loss of lean body mass occurs with age and this may contribute substantially to variability in BMI [41].

As a marker of visceral fat mass we also found WC to be associated with CHD for both men and women. As Asians and South Asians have a higher percentage of body fat than white people with same BMI and as they can also have increased abdominal obesity with a lower BMI, prediction of CHD or CHD risk factors from central or total obesity alone might be misleading in these regions [42,43], and the best and a sole obesity indicator for this group of population to assess CHD risk probably cannot be recommended. It is often suggested that, for future prediction of CHD use of both BMI and WC should provide a better result. Supporting this, recently Takahashi et al showed that using a combination of both WC and BMI was superior to using only one of these [44]. Wang et al suggested that BMI and WC, rather than WC alone, should be included in metabolic risk assessment for Asian population [45]. A WHO expert committee also suggested that where possible both WC and BMI should be measured in clinical practice and public health surveillance for Asian people [11].

Our results should be interpreted within the context of few limitations and strengths. We acknowledge that instead of selecting controls at the end of all cases, density sampling, or recruiting them at the time of each case selection could have provided a better approximation to the risk ratio. We would also like to mention that, due to small sample size we were not able to calculate the cut off points of BMI or other indicators. Determining precise cut offs is important as the risk assessment of CHD not only depends on the use of specific obesity measure, but also can vary widely based on the cut points used for each of them [44–47]. For example, WHO currently uses BMI cut points of 25 or higher to define overweight and 30 or higher for obesity [11]. But several studies have examined appropriate cut points to define overweight and obesity in Asian and South Asian populations and have argued for lowering BMI limits for these groups of population [44–52]. Despite these limitations, this study had several important strengths. One of the major strengths of the present study was the enrollment of only incident cases. Thus, our estimates of association were likely to be more reflective of risk of the development of the CHD, not of the duration of the CHD. Outcome misclassification was likely to have caused minimal error on our estimates of odds ratios as the definition of CHD was very specific. In this study anthropometric variables were directly measured by a trained health worker and were not self-reported or self-measured, which eliminated bias-related differential reporting and minimized measurement error.

The present study suggested that BMI, WC and WHtR values were all positively associated with risk of CHD. In addition, for women WHR was also strongly associated. This association persisted after adjustment for confounding factors. We conclude that for this group of population there was no single best obesity indicator. Since the measurement of WC and BMI are inexpensive and can be done easily without a time consuming complicated technique, we recommend that both BMI and WC should be included in the clinical and gradually in the community setting for CHD risk assessment in these high-risk South Asian populations. Instead of using just one of these parameters, use of both BMI and WC will increase the possibility of detecting CHD and thus a substantial amount of CHD mortality can be prevented.

## Figures and Tables

**Table 1:** Socio-demographic characteristics of the study participants by gender from BSMMU hospital, Bangladesh (N=390).

	Male (n=270)			Female (n=120)		

	Control (n=137)	Case (n=133)	P value^c^	Control (n=68)	Case (n=52)	P value^c^

Age^a^ (years)	53.2 ± 8.3	53.0 ± 8.3	0.839^d^	51.3 ± 8.4	52.6 ± 8.4	0.405^d^

**Age group (years)**						
30–39	6 (4.4)	5 (3.8)	0.994	5 (7.4)	3 (5.8)	0.701^e^
40–49	34 (24.8)	34 (25.6)		16 (23.5)	11 (21.2)	
50–59	64 (46.7)	62 (46.6)		37 (54.4)	26 (50.0)	
60–70	33 (24.1)	32 (24.1)		10 (14.7)	12 (23.1)	

**Marital status**						
Never married	0	0	0.017	2 (3.0)	0	0.095^e^
Currently married	127 (94.1)	130 (97.7)		46 (68.7)	44 (84.6)	
Widow	7 (5.2)	3 (2.26)		19 (28.4)	8 (15.4)	
Divorced	1 (0.7)	0		0	0	

**Residence**						
Urban	74 (54.4)	41 (30.8)	<0.001	17 (25.4)	5 (9.6)	0.020
Rural	62 (45.6)	92 (69.2)		47 (70.1)	47 (90.4)	

**Education**						
College or university	53 (39.0)	26 (19.5)	<0.001	1 (1.5)	2 (3.8)	0.006^e^
Secondary school	56 (41.1)	32 (24.1)		11 (16.4)	2 (3.8)	
Primary school	16 (11.8)	47 (35.3)		33 (49.3)	16 (30.8)	
None	11 (8.1)	28 (21.1)		22 (32.8)	32 (61.5)	

**Household income**^a^,^b^						
Mean (SD)	4374 ± 2560	4223 ± 1335	0.03^f^	3203 ± 2459	4289 ± 1387	<0.001^f^

BSMMU: Bangabandhu Sheikh Mujib Medical University Percentages are in parenthesis next to each count

Missing Values male: Marital Status=2, Residence=1, Education= 1, Household income= 1

Missing Values female: Marital Status=1, Residence=4, Education= 1, Household income= 2

a Mean ± Standard Deviation

b Per capita monthly household income in taka

c Pearson Chi square except where noted

d Independent t test

e Fisher’s exact tests

f Mann Whitney Rank test

**Table 2:** Anthr opometric indices and cardiovascular risk factors of the study participants by gender from BSMMU hospital, Bangladesh (N=390).

	Male (n=270)			Female (n=120)		

	Control (n=137)	Case (n=133)	P value^d^	Control (n=68)	Case (n=52)	P value^d^

**Smoking**						
Never	40 (29.6)	46 (34.6)	0.002	67 (100.0)	50 (96.2)	0.189
Current	26 (19.0)	56 (42.1)		0	2 (3.8)	
Former	69 (50.4)	42 (31.6)		0	0	

**Leisure time physical activity**						
Mild or Sedentary	123 (90.4)	130 (97.7)	0.018	55 (83.3)	45 (86.5)	0.797
Moderate to heavy	13 (9.6)	3 (2.3)		11 (16.7)	7 (13.5)	

**Active time physical activity**						
Mild or Sedentary	106 (77.9)	118 (89.4)	0.013	62 (95.4)	49 (94.2)	0.99
Moderate to heavy	30 (22.1)	14 (10.6)		3 (4.6)	3 (5.8)	

**Diabetes mellitus**						
No	120 (88.2)	95 (71.4)	0.001	49 (73.1)	33 (63.5)	0.319
Yes	16 (11.8)	38 (28.6)		18 (26.9)	19 (36.5)	

**Hypertension**						
No	85 (62.5)	44 (33.1)	<0.001	50 (74.6)	14 (26.9)	<0.000
Yes	51 (37.5)	89 (66.9)		17 (25.4)	38 (73.1)	

**Body mass index**^a^,^b^	24.4 ± 2.7	25.1 ± 2.0	0.013^e^	23.39 ± 2.37	25.3 ± 2.5	<0.001^e^

**Waist Circumference**^a^,^c^	86.4 ± 6.9	88.9 ± 8.7	0.009^e^	81.17 ± 11.37	86.1 ± 6.9	0.007^e^

**Waist hip ratio**^a^	0.92 ± 0.03	0.93 ± 0.03	0.171^e^	0.88 ± 0.05	0.91 ± 0.02	0.002^e^

**Waist height ratio**^a^	0.54 ± 0.04	0.55 ± 0.05	0.230^e^	0.52 ± 0.07	0.57 ± 0.04	0.001^e^

BSMMU: Bangabandhu Sheikh Mujib Medical University

Percentages are in parenthesis next to each count.

Missing Values male: Hypertension =1, Diabetes mellitus =1, smoking =2, physical activity =1, Body Mass Index=3, Waist Circumference=1, Waist hip ratio=1, Waist height ratio=2

Missing Values female: Hypertension =1, Diabetes mellitus =1, smoking =1, physical activity =2, Body Mass Index=2, Waist Circumference, Waist hip ratio, Waist height ratio=4

a Mean ± Standard Deviation

b Body Mass Index calculated as weight in kilogram/height in meter square

c Circumference in cm

d Pearson Chi square except where noted

e Independent t test

**Table 3:** Gender specific odds ratio with 95% confidence intervals of Coronary Heart Disease associated with one standard deviation change in each measure of obesity, BSMMU hospital, Bangladesh (N=390).

Measure	Male (n=270) OR (95% CI)	Female (n=120) OR (95% CI)

**Body mass index**		
Unadjusted	1.37 (1.06–1.76)	2.30 (1.49–3.56)
Multivariate Model 1^a^	1.69 (1.24–2.32)	2.64 (1.61–4.34)
Multivariate Model 2^b^	1.58 (1.13–2.23)	2.28 (1.27–4.07)

**Waist circumference**		
Unadjusted	1.39 (1.08–1.81)	1.80 (1.14–2.82)
Multivariate Model 1^a^	1.94 (1.40–2.70)	1.82 (1.12–2.95)
Multivariate Model 2^b^	1.88 (1.33–2.68)	1.55 (0.95–2.75)

**Waist-hip ratio**		
Unadjusted	1.18 (0.93–1.51)	1.89 (1.25–2.87)
Multivariate Model 1^a^	1.29 (0.97–1.71)	1.94 (1.23–3.07)
Multivariate Model 2^b^	1.25 (0.92–1.68)	2.12 (1.17–3.85)

**Waist-height ratio**		
Unadjusted	1.16 (0.91–1.48)	2.19 (1.34–3.56)
Multivariate Model 1^a^	1.32 (1.01–2.16)	2.32 (1.36–3.96)
Multivariate Model 2^b^	1.25 (0.98–2.01)	2.02 (1.01–4.3)

BSMMU=Bangabandhu Sheikh Mujib Medical University

OR, odds ratio; 95% CI, 95% confidence interval.

Body Mass Index calculated as weight in kilogram/height in meter square.

a Logistic regression controlling for age (in years), smoking (never smoker, former smoker, current smoker), education of the participant (college or university level education, secondary school, primary school and no schooling), physical activity level during leisure time (sedentary to mild versus moderate to strenuous), physical activity level during work time (sedentary to mild versus moderate to strenuous) and residence (urban versus rural).

b Additional adjustment for history of diabetes mellitus and history of hypertension.

**Table 4:** Odds Ratio with 95% confidence intervals of Coronary Heart Disease by quartiles of BMI, Waist circumference of male participants, BSMMU hospital, Bangladesh (N=270).

	Quartiles			
Body mass index	1^st^	2^nd^	3^rd^	4^th^
No of Cases/controls	19/49	40/21	32/35	41/30
Quartile cut off points	< 23.15	23.16–24.60	24.62–26.15	26.23–31.37
Unadjusted	Ref	4.12 (1.99–8.54)	2.36 (1.15–4.82)	4.07 (1.98–8.38)
Multivariate Model 2^a^	-	3.39 (1.44–8.0)	2.16 (0.95–4.94)	4.78 (2.0–11.44)
Multivariate Model 2^b^	-	3.04 (1.21–7.64)	2.28 (0.94–5.53)	4.01 (1.56–10.29)
**Waist circumference (cm)**				
No of Cases/controls	27/37	35/34	32/36	39/29
Quartile cut off points (cm)	< 82.70	83.00–86.70	87.00.0–91.50	92.4.1–120.00
Unadjusted	Ref	1.41 (0.71–2.80)	1.22 (0.61–2.42)	1.84 (0.92–3.68)
Multivariate Model 2^a^	-	1.60 (0.69–3.72)	1.57 (0.69–3.60)	3.23 (1.41–7.37)
Multivariate Model 2^b^	-	1.60 (0.64–3.97)	1.80 (0.73–4.43)	2.88 (1.17–7.09)

BSMMU: Bangabandhu Sheikh Mujib Medical University

OR, odds ratio; 95% CI, 95% confidence interval.

Body Mass Index calculated as weight in kilogram/height in meter square.

a Logistic regression controlling for age (in years), smoking (never smoker, former smoker, current smoker), education of the participant (college or university level education, secondary school, primary school and no schooling), physical activity level during leisure time (sedentary to mild versus moderate to strenuous), physical activity level during work time (sedentary to mild versus moderate to strenuous) and residence (urban versus rural).

b Additional adjustment for history of diabetes mellitus and history of hypertension.

**Table 5:** Odds Ratio with 95% confidence intervals of Coronary Heart Disease by quartiles of BMI, Waist circumference of female participants, BSMMU hospital, Bangladesh (N=120).

	Quartiles			
Body mass index	1^st^	2^nd^	3^rd^	4^th^
No of Case/controls	7/22	11/18	12/18	21/9
Quartile cut off points	< 22.15	22.19–24.14	24.32–26.27	26.33–30.90
Unadjusted	Ref	1.92 (0.62–5.97)	2.09 (0.68–6.43)	7.33 (2.31–23.27)
Multivariate Model 2^a^	-	2.73 (0.80–9.29)	2.71 (0.80–9.17)	9.45 (3.09–33.48)
Multivariate Model 2^b^	-	2.06 (0.49–8.55)	1.77 (0.42–7.55)	6.27 (1.33–26.52)
**Waist circumference (cm)**				
No of Cases/controls	7/22	11/19	17/13	14/13
Quartile cut off points (cm)	< 78.50	78.80–84.00	84.80–89.00	89.50–106.00
Unadjusted	Ref	1.82 (0.59–5.63)	4.11 (1.35–12.54)	3.38 (1.09–10.55)
Multivariate Model 2^a^	-	2.33 (0.69–7.87)	3.78 (1.19–12.09)	3.51 (1.03–12.00)
Multivariate Model 2^b^	-	1.19 (0.26–5.55)	3.61 (0.85–17.18)	2.08 (0.43–9.98)

BSMMU=Bangabandhu Sheikh Mujib Medical University

OR, odds ratio; 95% CI, 95% confidence interval.

Body Mass Index calculated as weight in kilogram/height in meter square.

a Logistic regression controlling for age (in years), smoking (never smoker, former smoker, current smoker), education of the participant (college or university level education, secondary school, primary school and no schooling), physical activity level during leisure time (sedentary to mild versus moderate to strenuous), physical activity level during work time (sedentary to mild versus moderate to strenuous) and residence (urban versus rural).

b Additional adjustment for history of diabetes mellitus and history of hypertension.
